# Ultrasensitive Diamond Cantilever‐Based Optical Microphone

**DOI:** 10.1002/advs.202516099

**Published:** 2025-11-03

**Authors:** Shen Tian, Chaonan Lin, Yingying Qiao, Mingyang Feng, Yang Gao, Kaijun Mu, Mingqi Jiao, Lei Li, Chongxin Shan

**Affiliations:** ^1^ Key Laboratory of Materials Physics Ministry of Education, School of Physics Zhengzhou University Zhengzhou 450001 China; ^2^ Laboratory of Zhongyuan Light Zhengzhou University Zhengzhou 450001 China; ^3^ College of Mechanical and Electrical Engineering Zhengzhou University of Light Industry Zhengzhou 450002 China; ^4^ Institute of Quantum Materials and Physics Henan Academy of Sciences Zhengzhou 450046 China

**Keywords:** cantilever, diamond, Fabry‐Perot interferometer, optical microphone

## Abstract

Microphones are critical for demanding scientific and industrial applications such as photoacoustic spectrometry, medical imaging, nondestructive testing, and trace gas detection. Achieving high sensitivity, low noise, and minimal detection levels in a compact device is a well‐known challenge. Herein, a novel diamond cantilever‐based optical microphone (DCOM) is reported using a Fabry‐Perot interferometric configuration. The DCOM achieves ultrahigh sensitivity (51.5 VPa^−1^) and a record detection level (0.018 µPa/$\sqrt{\mathrm{Hz}}$), placing it among the highest‐performing optical microphones reported to date. The outstanding performance of the DCOM can be attributed to the high quality factor and low intrinsic thermal noise of the diamond cantilever. Furthermore, the DCOM is applied in far‐field acoustic sensing and its long‐term stability is tested in corrosive environments. These results highlight the DCOM's potential for demanding industrial applications.

## Graphical abstract

The first demonstration that high quality diamond cantilevers function as high‐sensitivity acoustic transducers is reported, combining CVD diamond cantilevers and an extrinsic Fabry‐Perot optical configuration. High transduction efficiency yields record sensitivity (51.5 VPa^−1^) and MDP (0.018 µPa/$\sqrt{\mathrm{Hz}}$), enabling durable, low‐noise sensing for challenging industrial applications.

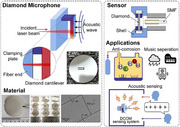

## 1 Introduction

Microphones^[^ ^1^, ^2^ ^]^ are transducers that convert airborne sounds into electrical signals. Traditional electrical microphones, including dynamic, piezoelectric, and condenser types,^[^ ^3^, ^4^, ^5^ ^]^ represent the state of the art in acoustic sensing technologies. These microphones typically exhibit one of two response patterns: a flat response across a wide frequency range for accurate sound reproduction, supporting civil applications such as voice interaction,^[^ ^6^ ^]^ hearing aids,^[^ ^7^ ^]^ and medical diagnostics;^[^ ^8^ ^]^ or a pronounced resonance peak for amplifying sounds at specific frequencies, enabling industrial applications such as photoacoustic spectroscopy,^[^ ^9^ ^]^ nondestructive testing,^[^ ^10^ ^]^ and gas leak detection.^[^ ^11^ ^]^ However, the latter type faces three key limitations in terms of the acoustic performance. First, its sensitivity is constrained by the energy dissipation of sound‐to‐electric conversion mechanisms.^[^ ^12^ ^]^ Second, its detection level is limited by various noise sources, including flicker noise, shot noise, and thermal noise, which impedes the detection of weak sounds. Third, its durability is compromised under harsh conditions, such as extreme temperatures and corrosive environments.

Optical microphones,^[^ ^13^, ^14^, ^15^, ^16^, ^17^ ^]^ which rely on highly sensitive photon detection and optically resonant structures, have begun to challenge their electrical counterparts. Various resonant structures, such as Fabry‐Perot (F‐P),^[^ ^18^ ^]^ microring^[^ ^19^ ^]^ and fiber Bragg grating,^[^ ^20^ ^]^ make it theoretically possible to achieve extremely high sensitivity. Among these, F‐P interferometric optical microphones,^[^ ^21^, ^22^, ^23^ ^]^ typically comprising a transduction element such as membrane, have garnered increasing attention due to their compact size and ease of fabrication. Membranes with thin thickness and high mechanical strength are crucial for exploring highly sensitive optical microphones. In the literature, various materials have been researched as membrane candidates, including metals,^[^ ^24^, ^25^ ^]^ polymers,^[^ ^26^ ^]^ and graphene.^[^ ^27^, ^28^ ^]^ However, diaphragms exhibit limited deformation amplitude and linearity due to radial stretching in response to acoustic pressure. To address this limitation, a cantilever structure has been investigated,^[^ ^29^, ^30^, ^31^ ^]^ offering acoustic‐induced deformation up to 112 times greater than that of a diaphragm under the same pressure. Therefore, the current trend in optical microphones has led to the development of highly sensitive microcantilevers. One strategy to increase the sensitivity of microphones involves developing longer, thinner cantilevers with higher quality. However, conventional materials exhibit limited mechanical strength. A long and thin cantilever may collapse due to gravity. Moreover, reducing the thickness to submicron levels can lead to an increase in the thermal oscillation amplitude, resulting in higher noise levels during deformation detection. Additionally, microcantilever tips are susceptible to corrosion and damage in harsh environments. It has proven challenging for a superthin microcantilever to maintain ultrahigh strength, high quality, sensitivity, and durability.

Diamond^[^ ^32^, ^33^ ^]^ is an exciting material with exceptional properties. It is the hardest and stiffest material with extraordinary chemical inertness, endowing it with vast potential applications in nanomechanics, optomechanics, and nanophotonics.^[^ ^34^ ^]^ The perfect mechanical and strength properties of diamonds, including Yong's modulus (>1100 GPa) and tensile stress (≈98 GPa), have led to a growing interest in recent works.^[^ ^35^, ^36^, ^37^, ^38^ ^]^ Notably, the resonant frequency of a diamond transducer is approximately two times that of silicon with identical geometry, and a diamond resonator can exhibit a quality factor (*Q*‐factor) exceeding 10^6^.^[^ ^39^ ^]^ Despite historically being considered a material that does not deform elastically, recent works have revealed its ability to achieve ultralarge elastic deformations.^[^ ^40^, ^41^, ^42^ ^]^ This breakthrough opens new possibilities for applications involving micro‐ and nanomechanical diamond transducers. However, the performance of diamond as an ultrasensitive acoustic transduction element in optical devices remains unexplored.

In this paper, we present a diamond cantilever‐based optical microphone (DCOM) for the first time. This concept effectively addresses the three aforementioned limitations of traditional microphones. We fabricated and thoroughly analyzed the acoustic performance of three distinct DCOMs with thicknesses of 10, 30, and 50 µm. For comparison, we also incorporated three optical microphones using stainless steel cantilevers of identical dimensions, measuring 10, 30, and 50 µm in thickness. The diamond cantilever‐based structure has yielded notable advantages in terms of sensitivity, frequency response, and minimum detectable acoustic pressure (MDP). This study has significant implications for advancing the field of acoustic sensing and paves the way for the development of high‐performance optical microphones with diverse applications.

## 2 Results and Discussion

### 2.1 Concept of a Diamond Cantilever‐Based Optical Microphone

**Figure 1:**
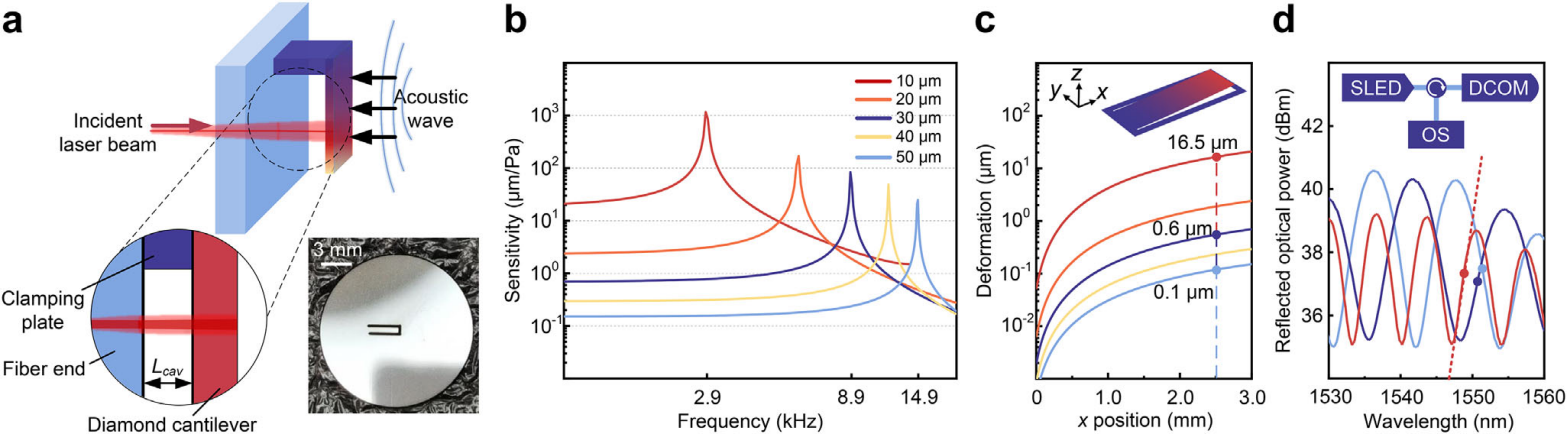
Concept of DCOM. a) Sensor schematic (*L*_cav_=length of F‐P cavity). The optical microphone comprises a diamond cantilever clamped on a fiber end. The color gradient represents the deformation amplitude. The photograph in the bottom right corner shows a circular diamond film with a cantilever structure before encapsulation in the sensor. Simulated mechanical sensitivity (b) and deformation amplitude (c) of the free end for diamond cantilevers with various thicknesses. Dimensions: cantilever length, 3 mm; width, 0.6 mm; thickness, 10–50 µm; gap to the film, 10 µm; acoustic pressure, 1 Pa; laser spot, *x*=2.5 mm. d) Quadrature working point (*Q*‐point) principle: SLED light is transmitted through a circulator, reflected by the DCOM, and detected by an optical spectrometer (OS). The optical sensitivity *S*_*i*_ of the sensor scales with the tangent of the curve (plotted on the spectrum of the cantilever with 10 µm thickness). The *Q*‐points are near 1549.0 nm (10 µm thickness), 1551.4 nm (30 µm thickness) and 1550.7 nm (50 µm thickness), respectively.

The transduction logic of the DCOM is outlined in **Figure** **1**. A cantilever is affixed to a cleaved optical fiber, thereby establishing an F‐P interferometric structure (Figure 1a). The incident laser beam is tuned to the wavelength λ, positioned within the linear response region (proximate to the quadrature point) of the microphone. When an external acoustic wave induces a pressure difference on the outer surface of the cantilever, the length of the F‐P cavity varies, resulting in a phase shift in the reflected light (see Experimental Section). The interference light is then detected by a photodiode. The magnitude of the phase shift is determined by the deformation flexibility of the cantilever (see Experimental Section). To optimize this amplitude, a cantilever structure is positioned at the center of the circular diaphragm, ensuring alignment with the point of maximal displacement amplitude. The mechanical sensitivity and first‐order resonant frequency of a series of diamond cantilevers graded by thickness are simulated (Figure 1b). Thinner cantilevers are inclined to deform under the same force, as demonstrated in Figure 1c, yet their lower resonant frequency constrains the bandwidth of their frequency response. To achieve balanced performance, we opt for 10, 30, and 50 µm thick cantilevers for the sensor design. The F‐P interference spectra within the DCOMs are shown in Figure 1d. A SLED light source was introduced into the F‐P cavity, yielding a fringe visibility of 4‐6 dB in the vicinity of the 1550 nm wavelength (see Section S3, Supporting Information).

The strong acousto‐optic transduction facilitated by the diamond cantilever creates opportunities to maximize sensitivity in three ways. First, diamond serves as an ideal transduction element due to its extraordinary mechanical strength and high *Q*‐factor. The diamond cantilever can be precisely tailored to be longer and thinner, achieving elevated sensitivity without compromising stability and frequency response (see Experimental Section). The *Q*‐factor, a key figure of merit for a sensitive mechanical transducer, represents the rate at which it absorbs or releases mechanical energy. Compared with other materials, diamond has the lowest intrinsic energy dissipation (*Q* ^−1^) due to its tightly bound lattice and excellent tribological properties, which maximize the transduction efficiency under the acoustic field.^[^ ^43^ ^]^ Second, the ultrahigh Young's modulus of diamond suppresses the thermal oscillation amplitude of the cantilever, thereby reducing the intrinsic thermal noise and improving the MDP (see Experimental Section). As the hardest known natural material, diamond can mitigate initial bending, a factor that makes microcantilevers more susceptible to spurious signals caused by temperature fluctuations. Third, the chemical inertness of diamond makes microcantilevers less susceptible to failure under conditions such as high temperatures, humidity, and corrosion. To achieve high performance and high reliability, it is desirable to develop all‐diamond structures compatible with the diamond cantilever for integration.

**Figure 2:**
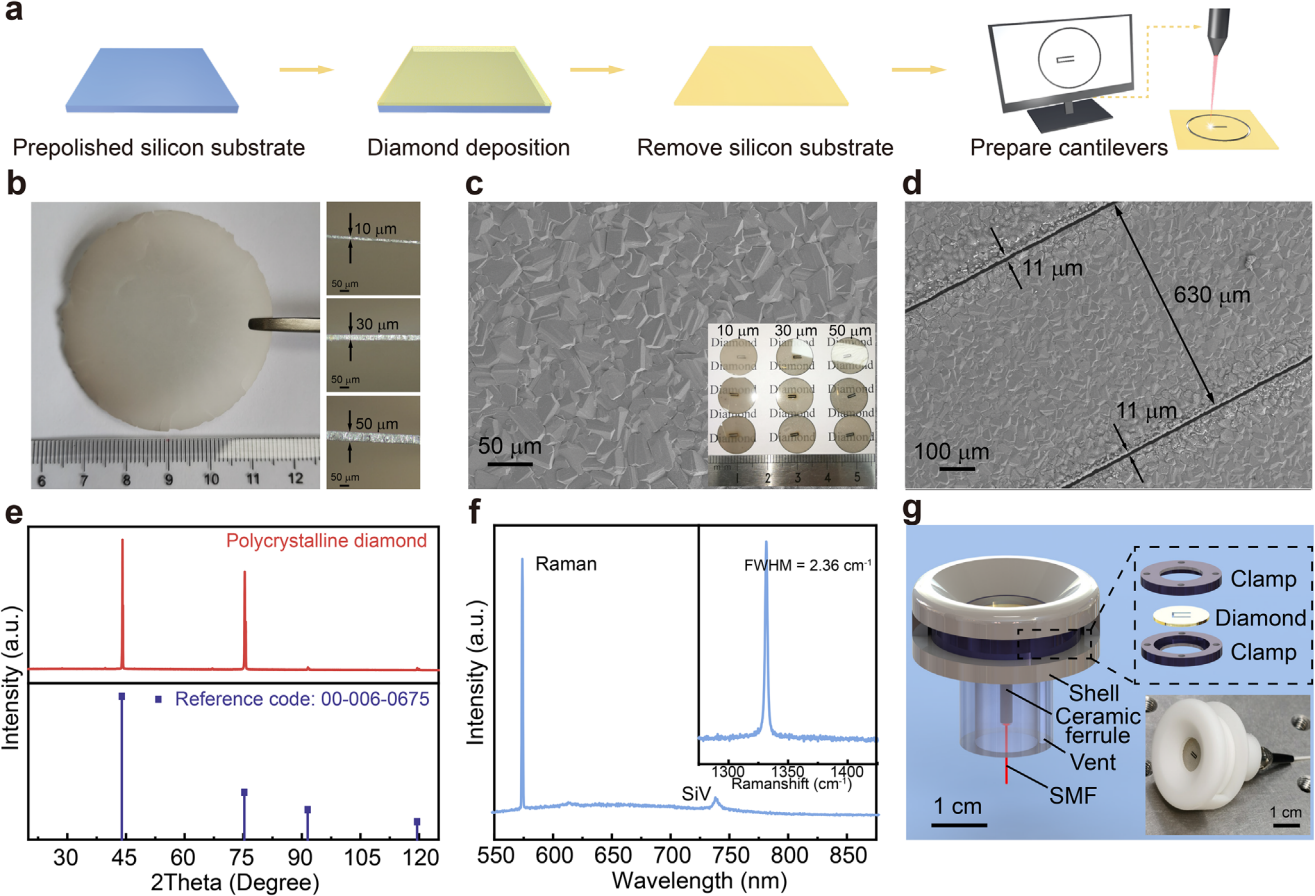
Fabrication process of the diamond cantilevers. a) Illustration of the fabrication process, including diamond film deposition and cantilever preparation. b) Large‐sized MPCVD diamond film with thicknesses *h*_1_ = 10 µm, *h*_2_ = 30 µm, and *h*_3_ = 50 µm. SEM images of the c) diamond film and d) cantilever; inset: prepared circular diamond membranes with cantilever structures. Membrane diameter, 12 mm. Cantilever length, 3 mm. e) XRD patterns and f) luminescence spectra of (c). g) Illustration of the assembly structure, comprising a ceramic ferrule and a diamond cantilever fixed by two ring clamps, both of which are encapsulated in an epoxy shell. A penetration vent is included to balance the air pressure. SMF, single‐mode fiber.

It is challenging to fabricate large‐sized thin diamond films and achieve precise cutting of this stiffest material. In this work, we solved these problems via the chemical vapor deposition (CVD) technique, fabricated diamond cantilevers with graded thicknesses and assembled them into DCOMs, as illustrated in **Figure** **2**. Figure 2a shows a schematic of the fabrication process for preparing cantilevers using ultrathin diamond self‐supporting films. Polycrystalline diamond films were grown on Si substrates using the microwave plasma CVD (MPCVD) technique (see Experimental Section). The fabricated large diamond films with thicknesses of 10, 30, and 50 µm are shown in Figure 2b. The laser was then utilized to prepare the desired cantilever geometry. A scanning electron microscopy (SEM) image of the diamond film is shown in Figure 2c. The grain size is uniform and dense, indicating that the prepared ultrathin diamond has formed a complete film and can be used for cantilever beam preparation. Figure 2d demonstrates the cantilever beam SEM image, with a width of 630 µm and a gap of 11 µm to the film. X‐ray diffraction (XRD) patterns and luminescence spectra were utilized to characterize the crystal structure of the polycrystalline diamond, as shown in Figure 2e,f. There are four XRD peaks located at 43.8°, 75.3°, 91.5°, and 119.8°, which can be attributed to diffraction from the (111), (220), (311), and (100) facets of diamond, respectively. There are only diamond Raman and Si color‐center peaks in the luminescence spectrum after 532 nm laser excitation, where the Si comes from the silicon substrates used for growth. The inset in Figure 2f shows the diamond Raman peak with a full width at half maximum (FWHM) of only 2.36 ${\mathrm{cm}}^{-1}$, and no peak from the non‐diamond phase can be observed, indicating the high crystalline quality of the prepared diamond. The high flatness and refractive index (2.4) of the diamond surface^[^ ^44^ ^]^ provide efficient confinement of light and distinct interferometric fringes within the DCOM, resulting in heightened optical sensitivity (*S*_*i*_). The assembly structure of the proposed DCOMs is depicted in Figure 2g, featuring distinct cantilever thicknesses (10, 30, and 50 µm) as the test group (see Experimental Section). Additionally, three sensors with stainless steel cantilevers and identical structures were fabricated for the comparison group (see Section S4, Supporting Information). The sensors were characterized in terms of their acoustic performance, as described below.

### 2.2 Sensor Characterization

**Figure 3:**
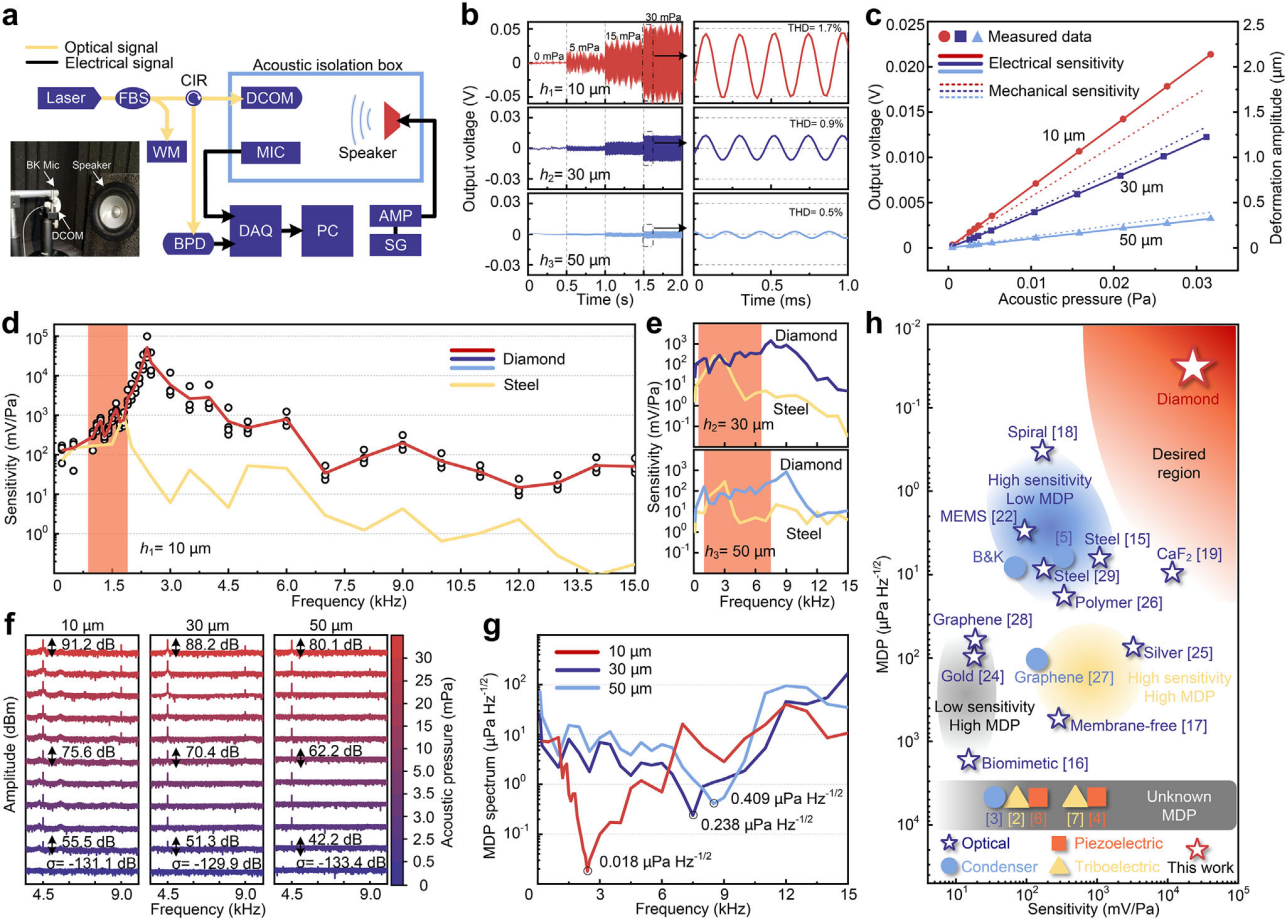
Sensor characterization and comparison. a) Acoustic sensing system setup. Photograph in the corner shows the arrangement inside the box. b) From top to bottom, output voltages of the DCOMs under acoustic pressures of 0, 5, 15, and 30 mPa. The right column shows a zoomed‐in response at 30 mPa pressure; maximum total harmonic distortion (THD)⩽ 1.7%. c) Linear fitting of output voltages as a function of applied pressure for 10 µm (red dotted line), 30 µm (navy square line) and 50 µm (blue triangle line) DCOMs; the dashed lines represent the deformation of the corresponding cantilevers. Frequency responses of d) 10 µm, (e, top) 30 µm, and (e, bottom) 50 µm DCOMs. Three sensors with stainless steel cantilevers were characterized as comparison groups (see Section S4, Supporting Information). f) Signal‐to‐noise ratio (SNR) spectra of (c). g) MDP spectra. Applied acoustic pressure *P* = 5 mPa, RBW Δ*f*=1 Hz. SNR values at the minima are 108.9, 86.5, and 81.7 dB for the 10, 30, and 50 µm DCOMs, respectively. h) Comparison of the sensitivity ($mV_{\mathrm{amp}}\mathrm{Pa}$ ^−1^) and MDP (µPa/$\sqrt{\mathrm{Hz}}$) of the 10 µm DCOM with existing microphones using various transduction mechanisms, including optical, piezoelectric, condenser, and triboelectric, shown in different colors. The commercial condenser microphone listed in this figure is B&K (47.8 $mV_{\mathrm{rms}}\mathrm{Pa}$ ^−1^), which is used as a sound pressure calibrator. The electrical sensitivity of all reported metrics is converted (see Section S6, Supporting Information). FBS, fiber splitter. CIR, circulator. WM, multi‐wavelength meter. BPD, balanced photodetector. AMP, power amplifier. SG, signal generator.

The sensor was illuminated by a laser and placed within an acoustic isolation box alongside a calibrated condenser microphone (see **Figure** **3**a and Experimental Section). Figure 3b presents the real‐time outputs of three DCOMs for various acoustic pressures at 4.5 kHz compared with the background noise. The amplitudes of the sinusoidal curves increase as the acoustic pressure arise, indicating that the sensor operates in the linear region and that the signals are free from distortion. Among the three sensors, the 10 µm DCOM exhibited a higher output voltage amplitude. To assess the acoustic performance of the sensors, measurements were conducted to determine their sensitivity, frequency response, and MDP.

#### 2.2.1 Sensitivity

We address the overall electrical sensitivity of the sensors, given by the ratio between the electrical output and the input acoustic pressure. In this work, this sensitivity strongly relies on the material properties, cantilever dimensions, and optical configurations (see Experimental Section and Section S2, Supporting Information). We demonstrated the output signal as a function of the applied acoustic pressure ranging from 0.5 to 30 mPa (Figure 3c). Due to the limitations of the acoustic isolation box and speaker, 0.5 mPa is the minimum pressure we can apply. The fitting curves of three DCOMs exhibited excellent linearity (*R* ^2^ = 0.999). Under identical test conditions, the thinner cantilevers exhibited greater sensitivity. The 10 µm DCOM achieved a sensitivity of 696.3 $mV_{\mathrm{amp}}\mathrm{Pa}$ ^−1^, or equivalently 492.4 $mV_{\mathrm{rms}}\mathrm{Pa}$ ^−1^ or ‐6.2 dBV (see Section S1, Supporting Information), surpassing the 392.2 $mV_{\mathrm{amp}}\mathrm{Pa}$ ^−1^ of the 30 µm DCOM and the 102.3 $mV_{\mathrm{amp}}\mathrm{Pa}$ ^−1^ of the 50 µm DCOM. The mechanical sensitivities (Δ*L*_cav_/Δ*P*_in_) of three diamond cantilevers were calculated as 56.7, 42.8, and 12.5 µmPa^−1^, respectively (see Section S4, Supporting Information). Overall, the DCOMs demonstrated 2 to 10 times the sensitivity of condenser microphones, highlighting their superior performance in acoustic sensing.

#### 2.2.2 Frequency Response

We characterized the frequency response of the 10 µm (Figure 3d), 30 µm and 50 µm (Figure 3e) DCOMs over a range from 100 Hz to 15 kHz. The 10 µm DCOM exhibited a flat bandwidth spanning from 800 Hz to 1.9 kHz, with an average sensitivity of 894.9 $mV_{\mathrm{amp}}\mathrm{Pa}$ ^−1^ at 1.5 kHz, far surpassing the sensitivity levels of the 30 and 50 µm DCOMs. Its resonance point was approximately 2.4 kHz, where it reached a sensitivity of 51.5 $V_{\mathrm{amp}}\mathrm{Pa}$ ^−1^. The phase response corresponding to Figure 3d is provided in Figure S7 (see Section S5, Supporting Information). In comparison, the 30 µm DCOM demonstrated a flat response from 500 Hz to 6.5 kHz, with an average sensitivity of 392.2 $mV_{\mathrm{amp}}\mathrm{Pa}$ ^−1^ at 4.5 kHz. The resonant frequency for this sensor was approximately 7.5 kHz, with a peak sensitivity of 1.4 $V_{\mathrm{amp}}\mathrm{Pa}$ ^−1^ at that frequency. Similarly, the 50 µm DCOM displayed a flat bandwidth from 1 to 8.5 kHz, with an average sensitivity of 102.3 $mV_{\mathrm{amp}}\mathrm{Pa}$ ^−1^. Its resonant frequency was near 9 kHz, achieving a sensitivity of 838.8 $mV_{\mathrm{amp}}\mathrm{Pa}$ ^−1^. For comparison, sensors with stainless steel cantilevers (yellow lines) were tested under the same conditions, showing much narrower bandwidths with resonances at 1.8, 2, and 3 kHz. The ultrahigh Young's modulus and low density of diamond result in a cantilever with a high resonant frequency and a wide frequency bandwidth, providing greater flexibility in the sensitivity‐bandwidth trade‐off.

#### 2.2.3 Minimum Detectable Acoustic Pressure

We conducted an analysis of the SNR and MDP of the proposed sensors. Three DCOMs exhibited remarkable SNRs of 91.2, 88.2, and 80.1 dB under an acoustic pressure of 30 mPa (63.5 dB SPL, Figure 3f), with a maximum THD below 1.7%. Notably, the 10 µm DCOM demonstrated an SNR of 108.9 dB and achieved the lowest reported MDP at 0.018 µPa/$\sqrt{\mathrm{Hz}}$ (measured at resonance, see Figure 3g and Experimental Section). In a 1‐Hz bandwidth at 4.5 kHz, the MDP is 0.83 µPa (‐27.6 dB SPL), yielding an estimated dynamic range of 91.2 dB. This exceptional performance can be attributed to the ultrahigh spring constant and low thermal noise properties of the diamond material. Moreover, the low MDP enables the sensor to detect minute acoustic signals, even in challenging industrial environments.

In this work, we achieved a combination of ultrahigh sensitivity and an outstanding detection limit owing to the unique properties of diamond materials. Compared with other microphone mechanisms, the DCOM confers a distinct advantage in terms of acoustic transduction, as depicted in the joint sensitivity‐MDP plot in Figure 3h and Table S1 (Supporting Information). Furthermore, the sensitivity could be further improved by increasing the length and decreasing the thickness of the diamond cantilever without introducing initial bending and thermal oscillation. The ultrahigh sensitivity and low MDP of the DCOM hold immense potential for advancing acoustic sensing applications. The 10 µm DCOM was applied for weak acoustic signal sensing, as described below.

### 2.3 Near‐Field and Far‐Field Acoustic Sensing

**Figure 4:**
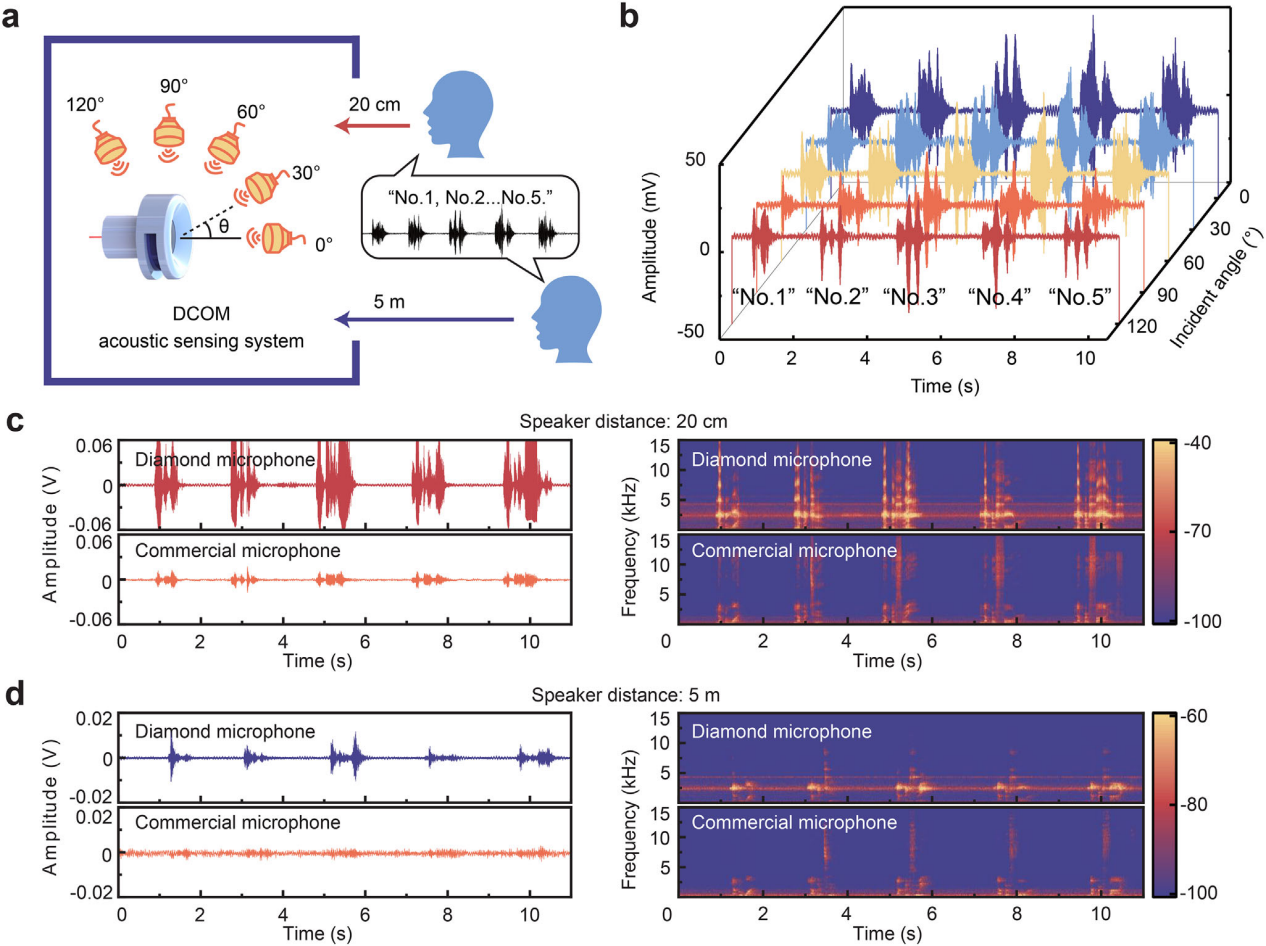
Application of the DCOM system in acoustic sensing. a) Schematic diagram of acoustic sensing at different incident angles and distances (20 cm and 5 m). b) Response curve of the sensor at 0–120°. Time‐domain and spectrogram diagrams of the signal reconstructed by the DCOM system at distances of c) 20 cm and d) 5 m, respectively.

The sensor was characterized by sound sources at different incident angles (in a sealed box) and distances (in an open box). The proposed DCOM exhibits cardioid directivity (see Section S6, Supporting Information) and produces varying acoustic sensing outcomes based on different incident angles. To verify the directional response characteristics of the DCOM, we employed an open‐source audio file containing the English phrases “Number one, number two, number three, number four, number five”, spoken by a male speaker. This audio file was played for the DCOM at 30° intervals within the horizontal plane, as indicated on the left of **Figure** **4**a. The output voltage responses of the sensor were meticulously recorded and are depicted in Figure 4b. Notably, the voltage amplitude decreases as the angle diminishes from 0°. The waterfall plot featured in Figure 4b underscores the efficacy of the proposed DCOM for directional acoustic sensing applications.

To evaluate the capability of DCOM to detect weak sounds, the aforementioned audio file was broadcast at distances of 20 cm and 5 m (including random environmental noise) from the sensor head, simulating near‐field and far‐field scenarios, respectively. To imitate practical scenarios, the tests are conducted in an open box, as depicted on the right of Figure 4a. Both the time‐domain and spectrogram diagrams of the DCOM‐reconstructed acoustic signals are depicted in Figure 4c, d, respectively. Due to the exceptional ultrahigh sensitivity and elevated SNR of the DCOM, the reconstructed signal exhibits high output amplitudes, corresponding to each phrase of the audio file. Notably, even at a distance of 5 m, the sensor system accurately portrays the distinctive characteristics of the uttered voice (Figure 4d). The exceptional sensitivity and low noise of the DCOM make it highly suitable for detecting weak acoustic signals in industrial environments.

### 2.4 Demonstration of Sensors in Harsh Environment

**Figure 5:**
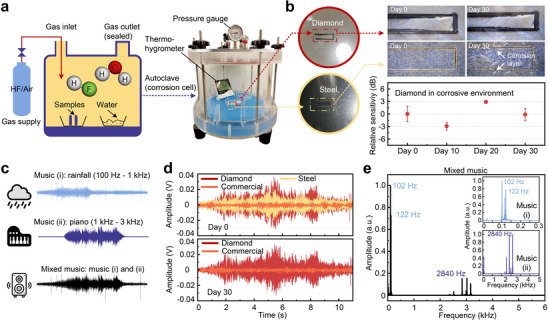
Demonstration of sensor performance in a corrosive environment and acoustic signal separation. a) Experimental setup for creating the corrosive environment. b) Optical images of cantilevers before and after 30 days in a sealed autoclave, along with sensitivity changes in the DCOM after exposure to corrosion. c) Setup for separating music segments. d) Reconstructed mixed music signals from diamond cantilever (red line), stainless steel cantilever (yellow line), and commercial microphone (orange line). e) Frequency spectra of the DCOM in response to mixed music after 30 days of exposure; insets: spectra for each music segment played individually.

The DCOM system demonstrates a remarkable ability to distinguish multiple sounds in harsh environments. **Figure** **5** demonstrates the long‐term durability of the diamond cantilever. In this test, both diamond and stainless steel cantilevers were placed inside a sealed autoclave filled with HF gas (see Figure 5a and Experimental Section). After 30 days, the surface of the diamond cantilever showed virtually no changes, whereas the stainless steel surface exhibited a corrosion layer, as shown in Figure 5b. The relative sensitivity of the DCOM changes within ±3 dB, highlighting that the diamond cantilever maintained stable performance in the corrosive environment, whereas the stainless steel failed due to corrosion.

To assess the capability of the sensors to separate overlapping acoustic signals, a test involving two different music segments was conducted. Figure 5c shows the features of two segments: i) rainfall sounds, concentrated in the range of 100 Hz to 1 kHz, and ii) piano music, concentrated in the range of 1 to 3 kHz. The reconstructed signals obtained from the sensors are displayed in Figure 5d, with a commercial condenser microphone used as a reference. Both the diamond and stainless steel optical microphones produced output voltage amplitudes noticeably higher than that of the commercial microphone. Notably, after 30 days in the corrosive environment, the maximum output voltage of the diamond cantilever decreased by only a few millivolts, whereas the stainless steel cantilever failed. The frequency response of the reconstructed signal collected by the 10 µm DCOM after 30 days in the corrosive environment is shown in Figure 5e. The lower frequency component is attributed to music (i), while the higher frequency component corresponds to music (ii). Furthermore, the DCOM output fluctuates by about ±0.5 mV over the 10–50 °C range (see Section S5, Supporting Information), indicating good stability against varying temperature. These tests confirmed that the diamond cantilever effectively recognized and separated the acoustic signals, even after extended exposure to harsh conditions.

## 3 Conclusion

In summary, this study has introduced a proven framework of an ultrasensitive optical microphone using a diamond cantilever. This framework leverages the advantages of a compact F‐P configuration and a superthin diamond cantilever with high quality, exceptional strength, stability, and durability. To overcome the challenges in the batch fabrication of diamond cantilevers, we utilized the CVD technique to produce large‐sized films with varying thicknesses and precisely cut them into identical membranes with cantilever structures. Crucially, the superior sensitivity reflects the positive interaction of two key factors: high energy transduction of diamond cantilever (large Δ*L*_cav_ per pascal) and an optimized F–P optical readout biased at quadrature. Using identical transducer geometry and optical structures, diamond cantilevers outperform stainless‐steel cantilevers, confirming that the material's energy transduction efficiency is the dominant contributor to the sensitivity gain. The use of diamond cantilevers as sensing elements brings forth highly efficient energy transduction, surpassing existing state of the art techniques. Resonant amplification of the diamond cantilever produces large tip motions that both yield the peak sensitivity of 51.5 $V_{\mathrm{amp}}\mathrm{Pa}$ ^−1^ and elevate the off‐resonance response, resulting in a high broadband average sensitivity of 894.9 $mV_{\mathrm{amp}}\mathrm{Pa}$ ^−1^ across the frequency band. Meanwhile, diamond's exceptional mechanical properties enable ultrathin cantilevers that maintain high *Q* and low Brownian noise, thereby lowering the overall noise floor and improving the MDP. The unique combination of high sensitivity and low detection limit sets DCOM apart in terms of acoustic performance. This is exemplified by the compelling results illustrated in Figure 3, where the 10 µm DCOM achieves a record MDP of 0.018 µPa/$\sqrt{\mathrm{Hz}}$. Moreover, the high resonant frequency of the diamond enables the 30 and 50 µm DCOMs to extend their bandwidths to meet various bandwidth requirements. Such a level of acoustic performance significantly outperforms that of current condenser or optical microphone approaches (Figure 3h).

The proposed framework exhibits significant potential and scalability in sensor performance. Reducing the cantilever thickness and increasing its length to enhance mechanical sensitivity may offer opportunities for greater sensitivity and improved MDP, potentially reaching orders of VPa^−1^ and nPa/$\sqrt{\mathrm{Hz}}$. Further research is needed to explore the performance of optical microphones using high‐quality diamond cantilevers with thicknesses of a few micrometers or even nanometers. The proposed framework also exhibits customization and flexibility in sensor design. In this study, sensors covering the voice spectrum below 10 kHz were demonstrated, striking a balance between heightened sensitivity and frequency response. However, improvements in the frequency response bandwidth can be achieved through cantilever geometry design. It is conceivable to cover the voice spectrum beyond 10 kHz for wideband acoustic applications by using shorter diamond cantilevers. This sensitivity‐bandwidth interplay, guided by theoretical principles, unlocks a spectrum of applications ranging from weak acoustic signal pickups to high SNR audio signal detection up to the 20 kHz regime. Finally, as depicted in Figure 5, the DCOM holds potential for a multitude of application scenarios, enabling cost‐effective, flexible, long‐range acoustic signal sensing in complex environments. As mentioned previously, the sensitivity and detection limit are crucial for detecting weak acoustic signals in industrial fields. Compared to current electrical methods, the DCOM combines ultrahigh sensitivity, the lowest detection limit, and immunity to electromagnetic interference, extending its applications in various industrial fields such as air‐leakage localization, photoacoustic gas detection, and non‐destructive testing. Furthermore, the extreme chemical inertness offered by the diamond cantilever opens the door to extending current detection capabilities, particularly in high‐temperature, high‐humidity, and corrosive conditions, while also ensuring the long‐term durability of diamond‐based devices.

In conclusion, this research provides a new versatile and generalized approach to high‐performance acoustic detection that has the potential to extend the capabilities of a wide range of industrial sensing technologies.

## 4 Experimental Section

### Sensitivity

The DCOM operated on the principle of “acoustic‐optical‐electrical” transduction logic. The overall electrical sensitivity, typically expressed in mVPa^−1^, was influenced by four key factors: sound propagation loss, mechanical sensitivity of the transduction element, optical signal interactions, and readout system (see Section S2, Supporting Information). In this work, the electrical sensitivity of the optical microphone was addressed, which was influenced mainly by the mechanical sensitivity and optical configuration when connected to an identical system. The mechanical sensitivity was calculated by a conversion factor (unit: mVµm^−1^, see Section S2, Supporting Information).

### Cantilever Dynamic Mechanism

For cantilever‐based optical microphones, the mechanical sensitivity (*S*_*m*_) of the transduction element (cantilever) was determined by the Euler‐Bernoulli equation (see Section S2, Supporting Information). For a singly clamped cantilever with a rectangular cross‐section, the moment of inertia *I*_*z*_ = *h* ^3^ *w*/12. The first‐order eigenfrequency ω_0_ can be expressed as:

(1) $$\omega_{0}=\frac{1.875^{2}}{L^{2}}\sqrt{\frac{\hat{E}I_{z}}{\rho S}}=\frac{1.875^{2}}{2\sqrt{3}}\frac{h}{L^{2}}\sqrt{\frac{\hat{E}}{\rho }}$$

where *L* represents the length of the cantilever. If the cantilever has a high width‐to‐height ratio (*w*/*h* > 5), $\hat{E}$ is replaced by $\hat{E}/\left(1-\sigma^{2}\right)$, where σ represents the Poisson's ratio of the cantilever material. For cantilevers with the same geometry, a larger $\hat{E}$ and smaller ρ result in a higher resonant frequency.

### Damping, Noise and MDP Measurement

Considering the damping effect of air, the motion of the cantilever could be simplified as a single mass model using a harmonic oscillator (see Section S2, Supporting Information). Assuming that a uniform surface stress Δ*P*_in_ over the entire area of the cantilever causes bending, the tip deflection of the cantilever Δ*z* can be expressed using Stoney's equation:

(2) $$\Delta z=\Delta L_{\mathrm{cav}}=\frac{3L^{2}\left(1-\sigma \right)}{\hat{E}h^{2}}\Delta P_{\mathrm{in}}$$

where the mechanical sensitivity of the material, denoted as *S*_*m*_, is calculated as Δ*L*_cav_/Δ*P*_in_. The sensitivity could be optimized by reducing the thickness and increasing the length of the cantilever.

The MDP was the pressure that yields an SNR of unity within a bandwidth Δ*f*. In the absence of other noise sources, the detection limit of a cantilever was ultimately constrained by its Brownian motion.^[^ ^45^, ^46^, ^47^ ^]^ The low thermal noise of the diamond cantilever contributed to an extreme acoustic detection limit (see Section S2, Supporting Information).

### F‐P Interferometric Optical Microphone

In a static F‐P cavity, the phase difference of the two beam interference was δ = 4π*L*_cav_/λ, and the optical sensitivity *S*_*i*_ of the F‐P interferometer was the variation in the reflected light intensity Δ*I*_*r*_ induced by the cavity length Δ*L*_cav_, which can be expressed as:

(3) $$S_{i}=\frac{\Delta I_{r}}{\Delta L_{\mathrm{cav}}}=\frac{8\pi \sqrt{\xi R_{1}R_{2}}}{\lambda }I_{i}\sin\frac{4\pi L_{\mathrm{cav}}}{\lambda }$$

when *L*_cav_ = (2n+1)λ/8, *S*_*i*_ is maximized, and the operating point of the sensor is called the *Q*‐point.

### Fabrication of the Diamond Film and Cantilever

Before growth, the Si (100) substrates were polished using diamond micropowders to increase the nucleation density. This was important for the preparation of high‐quality ultrathin diamond films. Next, the substrates were ultrasonically cleaned in acetone, methanol, and deionized water for 10 min each and then blow‐dried with high‐purity nitrogen. Hydrogen and methane with a total flow rate of 500 sccm were employed as the reactant gases for the growth of diamond. During the growth process, the substrate temperature was set at approximately 880 °C. The ${\mathrm{CH}}_{4}$ concentrations in the ${\mathrm{CH}}_{4}$/$H_{2}$ mixture were set to 3%. After growth, the Si substrates were removed using NaOH solution to obtain self‐supported ultrathin diamond films. The thicknesses of the films were regulated by controlling the growth time. After growth for 15, 22, and 35 h, the thicknesses of the obtained diamond films were 10, 30, and 50 µm, respectively.

### Sensor Design

The diamond cantilever was meticulously transferred and securely fixed into two ring clamps (Figure 2g). These clamps were housed within a 3D‐printed epoxy shell using UV adhesive. The ceramic fiber end was inserted into the shell and mounted on a five‐axis precision alignment platform. The F–P cavity length was tuned with the platform while monitoring the reflected spectrum on an optical spectrum analyzer (see Section S3, Supporting Information). A vent connecting the F‐P cavity and the outer environment helps balance the air pressure, thereby decreasing the extrinsic damping effect caused by air.

### Acoustic Sensing System Setup

The optical microphones were characterized in an acoustic isolation box (Figure 3a). Acoustic waves were generated by a commercial loudspeaker (CHR90, Markaudio), which was connected to a power amplifier (ATA 309, Aigtek) and a signal generator (294 waveform generator, 100 Ms^−1^, Fluke). The light beam was generated using a near‐infrared DFB laser (1550 ± 1 nm, Fitel, Japan), tuned by current and temperature controllers (LDX‐3525B, LDT‐5525B, ILX Lightwave), observed by a multi‐wavelength meter (Keysight 86120D), sent through an optical microphone, and finally detected using a photodetector (PDB450C, Thorlabs). The output voltage was collected using a DAQ (NI 9250, National Instrument) and presented on a PC for data processing. The received acoustic pressure was calibrated using a commercial condenser microphone (Type 4966, B&K, 47.8 $mV_{\mathrm{rms}}\mathrm{Pa}$ ^−1^) at the same position as the optical microphone. In the sensor characterization experiment, the distance between the acoustic source and the optical or condenser microphone was 20 cm. In the acoustic sensing and directivity experiment, both the DCOM and the commercial microphone were mounted on a fixed stage, while the sound source was rotated from 0 to 120 degrees around the fixed point in the acoustic field (Figure 4a; Figure S6, Supporting Information).

### Microphone Characteristic Measurement

Experiments were conducted on six optical microphones, i.e., three with diamond cantilevers of 10, 30, and 50 µm thickness and the others with stainless steel cantilevers of 10, 30, and 50 µm thickness under identical conditions. The applied sinusoidal acoustic wave had a range of 100 Hz to 15 kHz, with the acoustic pressure divided into ten steps and controlled by the power amplifier. In the test group, the diamond sensor head was initially connected. The wavelength of the laser was tuned to be near the *Q*‐point so that the output voltage exhibited the highest slope under the acoustic field. Next, a single‐frequency acoustic signal was applied via the signal generator, and the acoustic pressure was increased step by step through the power amplifier. The output voltages of the sensor and commercial microphone were recorded at each step of the acoustic pressure. To assess the response of the sensor under the acoustic wave, the acoustic frequency was varied and the aforementioned steps were repeated. As a comparison group, the same procedure was utilized for testing the stainless steel optical microphones. To minimize measurement errors, this characteristic experiment was repeated and four sets of data were collected for the 10 µm DCOM (Figure 3d).

### Demonstrations of Corrosive Resistance and Acoustic Signal Separation

Before testing, 10 µm cantilevers were photographed, and the diamond cantilever was then integrated into the DCOM sensing system (as described in Figure 3). Its sensitivity was measured at 1.5 kHz, with four sets of data collected and averaged to establish the Day 0 baseline. The cantilevers were placed in a sealed autoclave, alongside a petri dish containing distilled water (Figure 5a). An electronic thermohygrometer was also sealed inside to monitor the conditions. After 10 min of stabilization, the initial temperature (*T*_0_), and pressure (*P*_0_) were recorded. HF gas (1050 ppm), mixed with air, was introduced and pressurized to 0.04 MPa. After 30 min of stabilization, the temperature (*T*_1_) and pressure (*P*_1_) were recorded. The atmosphere inside the autoclave, consisting of air, HF, and water vapor, resulted in an HF concentration of approximately 287 ppm, which was calculated via the ideal gas law. The autoclave was placed outdoors in a shaded, ventilated area, where the water fully evaporated, maintaining a relative humidity of over 95%.

The cantilevers were removed after 10, 20, and 30 days for surface imaging, sensitivity characterization, and music signal separation testing. The same pressurization and testing steps were repeated. Relative sensitivity was quantified using the formula: *S*_*r*_ = 20log (*S*/*S*_0_), where *S* represents the measured sensitivity and *S*_0_ denotes the Day 0 average sensitivity.

## Conflict of Interest

The authors declare no conflict of interest.

## Supporting information

Supporting Information

## Data Availability

The data that support the findings of this study are available from the corresponding author upon reasonable request
