# Supplementary material for: Ultrasensitive Diamond Cantilever‐Based Optical Microphone
Source: Adv Sci (Weinh). 2025 Nov 3;13(3):e16099. doi: 10.1002/advs.202516099 (PMC12806201; doi:10.1002/advs.202516099)
Supplement: Supplementary file 1 — Supporting Information [file ADVS-13-e16099-s001.pdf]

Supporting Information for

Ultrasensitive diamond cantilever-based optical microphone

Shen Tian†, Chaonan Lin†, Yingying Qiao, Mingyang Feng, Yang Gao, Kaijun Mu, Mingqi Jiao, Lei Li*, Chongxin Shan*

*Corresponding author: Lei Li, lilei@zzu.edu.cn; Chongxin Shan, cxshan@zzu.edu.cn

†These authors contributed equally to this work.

This PDF file includes:

Supporting Text Section 1 to 6

Figure S1 to S7

Table S1

Text S1. Performance metrics

Here, we list the definitions of relevant microphone performance metrics addressed in the main article, namely sensitivity, frequency response, signal-to-noise ratio, and minimum detectable acoustic pressure.

Sensitivity: ratio between electrical output and applied acoustic pressure, typically expressed in mV/Pa or dBV for commercial microphones. For optical microphones, this overall sensitivity is a combination of electrical sensitivity, which depends on the mechanical sensitivity of the acoustic transduction element, the optical sensitivity of the optical configuration, and the system sensitivity of the optical readout system.

Frequency response: sensitivity curve relative to the applied acoustic frequency, which typically has a resonant frequency peak. The general flat region of the frequency response curve is below the resonant frequency.

Signal-to-noise ratio (SNR): ratio between the output in response to a reference signal (1 kHz at 1 Pa) and the noise level of the microphone.

Minimum detectable acoustic pressure (MDP): acoustic detection limit of a microphone, defined as the lowest pressure for a signal-to-noise ratio S/N=1 in a resolution bandwidth (RBW) Δf.

In this work, the calculation formula for electrical sensitivity has been unified, and we provide corresponding conversion methods from different previous literature sources[1]:

$$\begin{aligned} S_e &= \frac{\Delta V}{\Delta P}(\mathrm{mV_{rms}/Pa}) = \frac{\sqrt{2}\Delta V}{\Delta P}(\mathrm{mV_{amp}/Pa}) \\ &= \frac{2\sqrt{2}\Delta V}{\Delta P}(\mathrm{mV_{pp}/Pa}) = 20\log_{10}(\frac{\Delta V/\Delta P}{\Delta V_0/\Delta P_0})(\mathrm{dBV}) \end{aligned} \tag{S1}$$

where ΔV represents the root-mean-square of the output voltage, with units in RMS, amplitude, and peak-to-peak denoted as mVrms, mVamp, and mVpp, respectively. V_0 is the reference voltage (1 V) at the reference pressure P_0 (1 Pa), defined as 0 dBV. Previous works often use peak-to-peak or amplitude values of voltage for calculating sensitivity, while commercial microphones are generally

calibrated with RMS values and in dBV. We have unified the units as mVamp for ease of general comparison.

Text S2. Transduction mechanism and modeling

Overall sensitivity: A typical structure of an optical microphone is shown in **Figure** S1. In this work, we address the electrical sensitivity, which strongly depends on the material properties, cantilever dimensions, and optical configurations. The electrical sensitivity S_e of the diamond cantilever-based optical microphone (DCOM) can be expressed as:

$$S_e = \frac{\Delta V}{\Delta P} = \frac{\Delta P_{\text{in}}}{\Delta P} \frac{\Delta L_{\text{cav}}}{\Delta P_{\text{in}}} \frac{\Delta I_r}{\Delta L_{\text{cav}}} \frac{\Delta V}{\Delta I_r} \tag{S2}$$

where V represents the output voltage of the sensing system, P represents the pressure generated by the acoustic source, P_{in} denotes the pressure difference between the two sides of the cantilever, L_{cav} represents the Fabry-Perot (F-P) cavity length, and I_r represents the reflected light intensity. Notably, in this study, the characteristics of the DCOM are tested in the near-field, so the propagation loss of the acoustic wave $\Delta P_{\text{in}}/\Delta P$ can be neglected. $\Delta L_{\text{cav}}/\Delta P_{\text{in}}$ indicates the mechanical sensitivity of the diamond material (S_m), $\Delta I_r/\Delta L_{\text{cav}}$ indicates the sensitivity (S_i) of F-P interferometry, and $\Delta V/\Delta I_r$ depends on the amplifier and the response of the photodetector in the readout system.

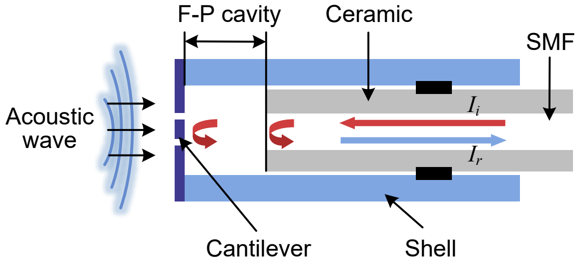

Figure S1: Optical microphone modeling. Schematic of cantilever-based F-P optical microphone.

Acousto-mechanical transduction modeling: Two typical models, the string model and the beam model, are applied to understand the dynamic mechanism of the transduction element. The string model is typically used for materials such as stainless steel, where residual stress during film processing or tensile stress applied during clamping serves as the main source of restoring force. In the case of monocrystalline diamond cantilevers, which are flexural rigid beams with rectangular

cross-sections, bending stress dominates (**Figure** S2). With appropriate simplifications for the thin-beam approximation and assuming no damping, the motion is determined by the Euler-Bernoulli equation[2]:

$$F_d = \frac{\partial^2 D(x,t)}{\partial t^2}\rho S + \frac{\partial^4 D(x,t)}{\partial x^4}\widehat{E} I_x \tag{S3}$$

where $D(x,t)$ denotes the displacement Δz in the z-axis direction due to the drive force (F_d), ρ denotes the mass density, and $S = wh$ denotes the cross-sectional area, where w and h represent the width and height of the cantilever, respectively. Additionally, $\widehat{E}$ denotes Young's modulus, and I_x denotes the moment of inertia of the cantilever. The solution to this differential equation describes the beam deformation as a harmonic motion with position-dependent and time-dependent terms, $D(x,t) = D_n(x)\exp(-\mathrm{i}\omega_n t)$, where ω_n represents the motion frequency and n denotes the modal number. In this work, we address the first-order resonant frequency of the cantilever.

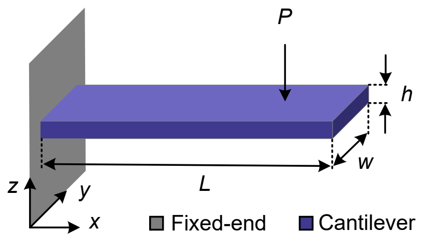

Figure S2: Cantilever modeling. Schematic of a simple cantilever geometry.

Considering the damping effect, the dynamic model can be simplified as a single mass model using a harmonic oscillator:

$$m_{\mathrm{eff}}\ddot{z} + \beta\dot{z} + k_{\mathrm{eff}} z = F\cos(\omega t) \tag{S4}$$

where m_{eff} represents the effective mass of the cantilever, β is the damping constant, k_{eff} is the effective spring constant, and $F\cos(\omega t)$ denotes the sinusoidal force caused by the acoustic wave. The intrinsic damping of the cantilever represents the energy dissipation mechanism[3], given by $\beta = \sqrt{k_{\mathrm{eff}} m_{\mathrm{eff}}}/Q_c$, where Q_c is the quality (Q) factor of the cantilever. The Q-factor is expressed as $Q_c = 2\pi W_0/\Delta W$, where W_0 represents the stored vibrational energy and ΔW represents the total energy dissipation per vibration cycle.

Extrinsic damping is caused by the medium and sensor geometry. For a cantilever clamped on the shell of an F-P sensor, when the cantilever oscillates, it displaces the gas molecules around it, resulting in a corresponding change in the volume of the F-P cavity[4]. The damping effect of

air introduces an additional component to the effective mass and spring constant. In this case, the effective spring constant and mass of the cantilever are as follows:

$$k_{\text{eff}} = \frac{2}{3}Ew(\frac{t_c}{L})^3 + \frac{2\kappa S^2 P}{5V}, m_{\text{eff}} = 0.647m + \frac{VM}{RT}P \tag{S5}$$

The solution $z(\omega)$ gives the amplitude of the cantilever as:

$$A_z(\omega) = \sqrt{z(\omega)z^*(\omega)} = \frac{F}{m_{\text{eff}}\sqrt{(\omega_0^2 - \omega^2)^2 - (\omega\beta/m_{\text{eff}})^2}} \tag{S6}$$

For force sensing applications, a cantilever's sensitivity is ultimately constrained by its thermally driven motion, with a noise-limited minimum pressure P_{min} [5, 6]:

$$P_{\text{min}} = \frac{P_{\text{in}}}{10^{\frac{\text{SNR}}{20}} \times \sqrt{\text{RBW}}} \tag{S7}$$

where SNR represents the signal-to-noise ratio in decibels, and RBW is the resolution bandwidth. The comprehensive noise profile includes factors such as shot noise, thermal noise, detector noise and DAQ noise. In this work, optical microphones were characterized under identical conditions, highlighting the predominant influence of noise originating from the thermal oscillation amplitude of the cantilever. In thermal equilibrium, the mean square displacement z_{rms} is related to the spring constant through the equipartition theorem, $\frac{1}{2}k_{\text{eff}}z_{\text{rms}}^2 = \frac{1}{2}k_B T$, where T is the temperature, and k_B is the Boltzmann constant. As per **Equation** S5, the ultrahigh Young's modulus of the diamond cantilever suppresses thermal oscillation under temperature variation, thereby providing low thermal noise.

Mechanical-optical modeling: The DCOM comprises a Fabry-Perot (F-P) cavity, a 3D-printed epoxy shell, and a single-mode fiber. The F-P cavity is enclosed within the sensor shell, which is formed by two reflective surfaces: the fiber end and the inner surface of the diamond film (with a cantilever structure in the middle). The interference model can be simplified as a two-beam interferometer, as depicted in **Figure** S3a.

The incident light can be reflected by both the diamond cantilever and the optical fiber end, and the reflective interference intensity I_r can be expressed as:

$$I_r = I_i(R_1 + \xi R_2 - 2\sqrt{\xi R_1 R_2}\cos\delta) \tag{S8}$$

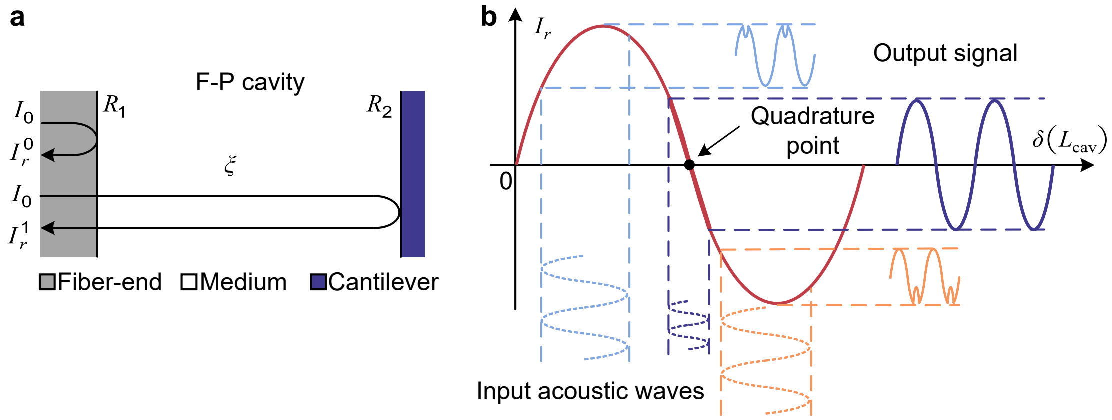

Figure S3: F-P interferometer modeling. (a) Schematic of F-P interference in DCOM. (b) Illustration of the linear response region and quadrature point.

where I_i is the intensity of the incident light; R_1 and R_2 are the reflectivity values of the fiber end and diamond cantilever, respectively; δ is the phase difference; and ξ is the optical coupling coefficient, which is dependent on the length of the F-P cavity and the wavelength of light. ξ can be expressed as:

$$\xi = \frac{4[1 + (\frac{2\lambda L_{\text{cav}}}{\pi n_0 \omega_0{}^2})^2]}{[2 + (\frac{2\lambda L_{\text{cav}}}{\pi n_0 \omega_0{}^2})^2]^2} \tag{S9}$$

where λ (1550 nm) denotes the wavelength of the incident laser source, L_{cav} denotes the length of the F-P cavity, n_0 denotes the refractive index of the air medium within the cavity, n_0=1, and ω_0 denotes the $1/e^2$ beam radius. According to the fringe visibility in Figure 1d of the main text, we calculated the reflectivity of the two surfaces in the F-P cavity. For the 10 µm DCOM, R_1 and ξR_2 were 2.5% and 17.2%, respectively. For the 30 µm DCOM, these values were 2.8% and 17.1%, and for the 50 µm DCOM, they were 3.4% and 17.0%.

In a static F-P cavity, the phase difference of the two beam interference is δ=$4\pi L_{\text{cav}}/\lambda$, and the optical sensitivity S_i of the F-P interferometer is the variation in the reflected light intensity ΔI_r induced by the cavity length ΔL_{cav}, which can be expressed as:

$$S_i = \frac{\Delta I_r}{\Delta L_{\text{cav}}} = \frac{8\pi\sqrt{\xi R_1 R_2}}{\lambda} I_i \sin\frac{4\pi L_{\text{cav}}}{\lambda} \tag{S10}$$

When the operating wavelength is set at the quadrature (Q) point of the cavity where the interference fringe slope is steepest (Figure S3b), the F-P interferometer responds approximately linearly and achieves its maximum optical sensitivity (navy curve). If the wavelength drifts to a fringe peak

or dip, both sensitivity and linearity fall off sharply (blue and orange curves). Therefore, during measurements the operating wavelength should be maintained near the Q-point to preserve a high, linear readout. When L_{cav}=(2n+1)λ/8, S_i is maximized, and the operating point of the sensor is near Q-point. The reflected light of the optical microphone in the acoustic field can be expressed as:

$$I_r = 2I_0[1 + \gamma\cos(\frac{4\pi(L_{cav} + \Delta L_{cav})}{\lambda} + \pi)] \tag{S11}$$

Optical-electrical modeling: The system sensitivity is mainly determined by the response of the photodiode. The response of balanced PD (PDB450C, Thorlabs) can be expressed as:

$$\Delta V = \Delta I_r \times \mathrm{R}(\lambda) \times \mathrm{G} \tag{S12}$$

where R(λ) denotes the responsivity of the photodiode at a given wavelength and G denotes the transimpedance gain of the PD output. The sensor is connected to the sensing system, and the relationship between the deformation of the diamond cantilever (Δz) and the output voltage of the photodetector (ΔV_r) can be expressed by the following equation:

$$\Delta z = \frac{\lambda(R_1 + \xi R_2)}{8\pi V_0\sqrt{\xi R_1 R_2}}\Delta V_r \tag{S13}$$

where V_0 is the output voltage from the PD at the Q-point. For the three DCOMs, the output voltages were -1.61 mV_{rms}, 1.15 mV_{rms}, and -0.96 mV_{rms}, respectively. Using **Equation** S13, the conversion factor from overall electrical sensitivity (mV/Pa) to mechanical sensitivity (µm/Pa) was calculated as 12.29 mV/µm, 9.16 mV/µm, and 8.20 mV/µm for the 10, 30, and 50 µm DCOMs, respectively.

Text S3. Additional data on the measurement of F-P interference

The experimental setup of F-P interference is shown in **Figure** S4a. Incident light from an SLED source (1550 ± 30 nm) was transmitted through a circulator, reflected by the sensor head, and detected by a fiber Bragg grating interrogation analyzer (FBGA, Bayspec). The FBGA has a frequency response time of 5 kHz and is connected to the PC via a USB interface. The use of the FBGA provides real-time and high-speed measurements of the F-P interference spectrum.

Fringe visibility, defined by $V = (I_{max} - I_{min})/(I_{max} + I_{min})$, represents the optical interference within the optical microphone, where I_{max} and I_{min} are the maximum and minimum intensities of

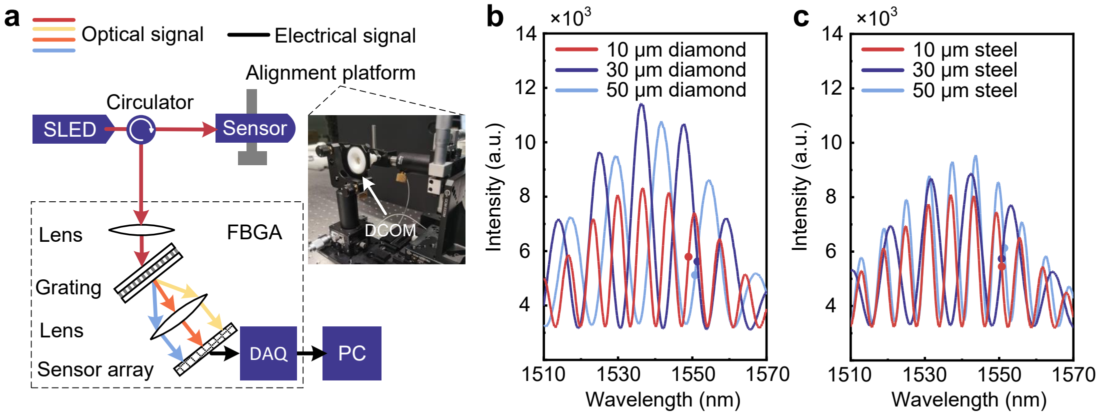

Figure S4: Measurement of F-P interference. (a) Experimental setup; inset: photograph of the alignment platform. (b) Interference spectra of the proposed DCOMs. The Q-points of the 10 μm (1549.0 nm, red dot), 30 μm (1551.4 nm, navy dot) and 50 μm (1550.7 nm, blue dot) cantilevers are marked on the curves. (c) Interference spectra of sensors using stainless steel cantilevers.

reflected light, respectively. Following the assembly of the cantilever and shell, all the fabricated sensors mentioned in the main article underwent testing for fringe visibility. The ceramic ferrule in the shell was adjusted to reach maximum fringe visibility. Figure S4b and S4c present the interference spectra of the 10 μm (red lines), 30 μm (navy lines) and 50 μm (blue lines) cantilevers, respectively. As depicted in Figure S4b, the spectral combs of the 10 μm, 30 μm and 50 μm DCOMs exhibit similar fringe visibility (approximately 6 dB) near 1540 nm. Considering the use of a 1550 nm DFB laser in the sensor characterization section, we estimated the Q-points Q_1 (1549.0 nm), Q_2 (1551.4 nm) and Q_3 (1550.7 nm) near 1550 nm, as depicted in Figure S4b and Figure 1d of the main article. Moreover, in Figure S4c, sensors using stainless steel cantilevers exhibit a similar fringe visibility of 5 dB, with the estimated Q-points Q_4 (1550.7 nm), Q_5 (1550.7 nm) and Q_6 (1551.0 nm).

In the sensor characterization experiment, the operating wavelength of the DFB laser (1550 ± 1 nm, Figure 3a of the main article) was adjusted to match these Q-points, so that the output voltage has the highest slope in response to varying acoustic pressure. At this working point the optical sensitivity S_i is maximized.

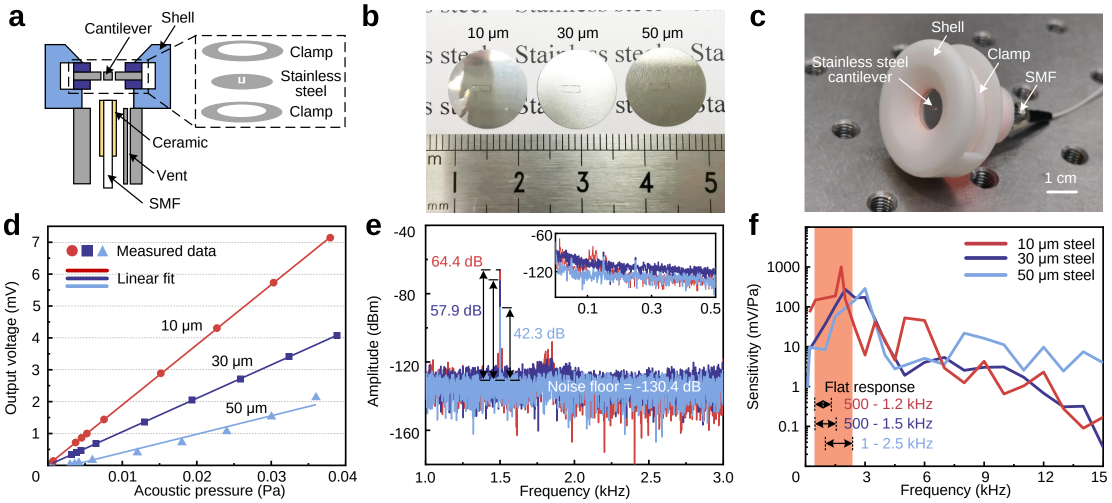

Figure S5: Sensor design and characterization. (a) Schematic of the assembly structure. (b) Photograph of micro-machined 10 μm, 30 μm and 50 μm stainless steel membranes with cantilever structures. Cantilever length, 3 mm. Cantilever width, 600 μm. (c) Illustration of the assembly sensor. (d) Linear fitting of output voltages as a function of applied pressure for 10 μm (red dotted line), 30 μm (navy square line) and 50 μm (blue triangle line) sensors. Acoustic frequency, 1.5 kHz. (e) SNR spectra of (d) under an acoustic pressure of 5 mPa; inset: zoomed-in low-frequency noise ranging from 0 to 0.5 kHz. (f) Frequency response.

Text S4. Additional data on the comparison group

We fabricated three optical microphones with 10 μm, 30 μm and 50 μm stainless steel cantilevers as a comparison group. The sensor structure, shown in **Figure** S5a, is identical to the proposed DCOMs (Figure 2g in the main article). The stainless steel membrane with a cantilever structure (Figure S5b) is secured by two clamps and enclosed in the shell. The assembled sensor is depicted in Figure S5c.

The performance of the stainless steel sensors was tested under identical procedures and conditions after the DCOMs were tested. We demonstrated the output signal as a function of the 1.5 kHz applied acoustic pressure ranging from 0.5 to 30 mPa (Figure S5d). The 10 μm sensor reached a sensitivity level of 188.4 mV_{amp}/Pa, surpassing the 30 μm and 50 μm sensors, which exhibited sensitivities of 105.0 mV_{amp}/Pa and 57.8 mV_{amp}/Pa, respectively. Notably, the fitting curves of the 50 μm sensor exhibited limited linearity (R^2=0.987), especially under pressures less than 10 mPa, demonstrating the limited sensitivity of the 50 μm stainless steel cantilever for weak acoustic signal sensing. Figure S5e displays the SNR spectra detected by the 10 μm (red line), 30 μm

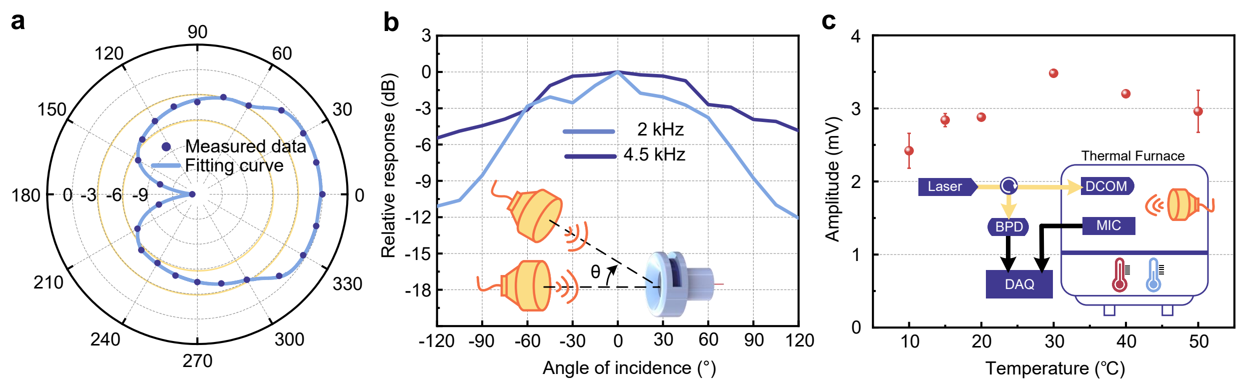

Figure S6: Directivity and stability of the DCOM. (a) Cardioid directivity diagram of the DCOM at 4.5 kHz. (b) Directional response at selected frequencies; inset: schematic of the directional response measurement. A speaker was placed 20 cm from the DCOM at 0°, and sensitivity was measured at 15° incremental angular intervals. (c) Output response of the DCOM under varying temperature; inset: schematic of the temperature stability test.

(navy line) and 50 μm (blue line) sensors under 5 mPa acoustic pressure. An SNR of 64.4 dB is observed at a frequency of 1.5 kHz, which corresponds to an MDP of 3.0 $\mu Pa/\sqrt{Hz}$ at a 1 Hz resolution bandwidth. The frequency response of the sensors is shown in Figure S5f, Figure 3d and 3e of the main article. Figure S5f shows the 10 μm sensor with a bandwidth of 500 Hz to 1.2 kHz and a resonant frequency of 1.8 kHz. The 30 μm sensor spans 500 Hz to 1.5 kHz with a 2.0 kHz resonant frequency, while the 50 μm sensor covers 1 kHz to 2.5 kHz, with a resonant frequency of 3.0 kHz. Compared with the DCOMs, stainless steel sensors have significantly lower sensitivity and a narrower response bandwidth.

Text S5. Additional data on the DCOM performance

Directivity. The concept of directivity, where the sensitivity of an acoustic sensor depends on the incident angle of acoustic waves, is a distinctive attribute predominantly determined by the size and geometry of the sensor. The directivity of the developed DCOM can be quantified by the expression: $D = 20\lg(S/S_0)$, where S represents the tested sensitivity and S_0 denotes the maximum sensitivity, both measured during the directivity experiment of the sensor (**Figure** S6).

In the experimental setup, the DCOM and commercial microphone were mounted on a fixed stage, while the speaker was positioned at a distance of 20 cm from the DCOM. All these sensors and speaker were enclosed in a sealed acoustic isolation box. The speaker rotated around a fixed

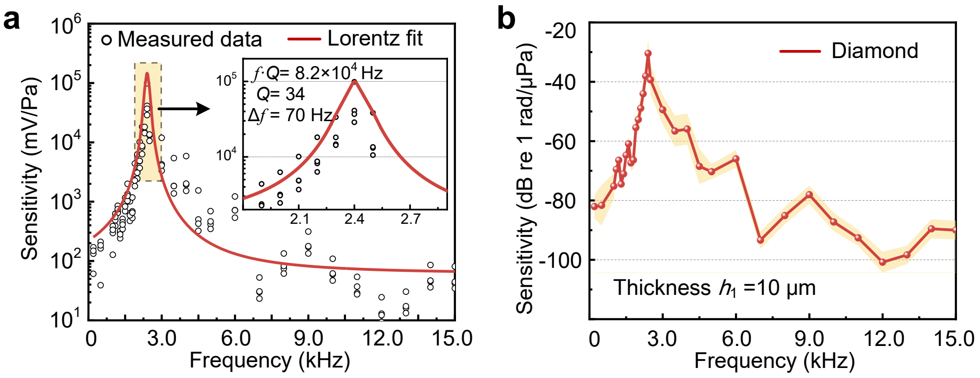

Figure S7: Frequency response of the DCOM. (a) Lorentz fit; measured quality factor Q=34 and product $f\cdot Q$=8.2×10^4 Hz. (b) Phase response; the yellow shaded region indicates the measurement uncertainty.

point within the acoustic field, and the sensitivity of the sensor was recorded at 15° intervals. The recorded data form a directional polar diagram, as depicted in Figure S6a, showcasing a cardioid response curve at an acoustic frequency of 4.5 kHz. This curve illustrates the proposed sensor's directional response exceeding -3 dB within the ±75° range and maintaining a response beyond -6 dB within approximately ±135°. Further investigations highlight the sensor's directivity under distinct frequencies, as demonstrated in Figure S6b. Evidently, the acoustic sensor exhibits appreciable directional response within ±60° across diverse acoustic frequencies.

Temperature stability. We evaluated temperature stability by placing the DCOM, B&K microphone, and the speaker inside a thermal furnace (see Figure S6c). The DCOM response to a 4.5 kHz, 30 mPa acoustic wave was measured at 10, 15, 20, 30, 40 and 50 °C. Each data point is the average of five independent measurements; error bars indicate the standard deviation. Over the tested temperature, the DCOM output varied by about ±0.5 mV, demonstrating good stability under these temperature conditions. Note that the current sensor uses a resin housing, which limits the upper-temperature testing to avoid packaging damage.

We calculated the estimated Q-factor of the DCOM using Lorentz fit, as depicted in **Figure** S7a. Figure S7b shows the converted phase response of Figure 3d in the main text.

Text S6. Sensitivity and MDP comparison

The performance metrics compared in the main article are listed in **Table** S1. Table S1 shows that the 10 μm DCOM offers markedly higher sensitivity and a much lower MDP than typical capacitive,

piezoelectric, and optical microphones. By comparison, most microphones report sensitivities of only tens to a few hundred mV/Pa and MDPs on the order of tens of μPa/$\sqrt{\text{Hz}}$ or higher; therefore, the DCOM's sensitivity and detection limit are improved by orders of magnitude in many cases.

Table S1: Sensor comparison. Comparison of the sensitivity (mV_{amp}/Pa) and MDP (µPa/$\sqrt{Hz}$) of the 10 µm DCOM with existing microphones using various signal transductions.

**Journal** [Ref. in main article] or commercial mic. (Company)	**Transduction mechanism**	**Materials/ structures**	**Sensitivity**	**MDP**
Adv. Mater. **34**, 2109545 (2022) [3]	Condenser	Polymer	31.7	NA
ACS Appl. Mater. Interfaces. **9**, 1237-1246 (2017) [27]	Condenser	Graphene	141	100
J. Microelectromech. S. **24**, 241-248 (2015) [5]	Condenser	Silicon	300*	6
Type 4966 (B&K, Denmark)	Condenser	Stainless steel	67.2	8
Sci. Adv. **7**, eabe5683 (2021) [6]	Piezoelectric	PZT membrane	73.5*	NA
Nat. Commun. **7**:11108 (2016) [4]	Piezoelectric	Polymer/Nanofiber web	376.2*	NA
Sci. Adv. **3**, eaas8772 (2018) [2]	Triboelectric	Silver nanowire	49.5*	NA
Sci. Robot. **3**, eaat2516 (2018) [7]	Triboelectric	Polymer	369.1	NA
IEEE Sens. J. **21**, 17882-17888 (2021) [24]	Optical	Gold membrane	17.8*	95.3
J. Lightw. Technol. **36**, 5650–5655 (2018) [26]	Optical	Polymer membrane	342.78*	17.9
IEEE Photonic. Tech. L. **25**, 932-935 (2013) [28]	Optical	Graphene membrane	18.6	60
J. Lightw. Technol. **36**, 5224–5229 (2018) [22]	Optical	Corrugated MEMS membrane	91.78*	2.97
IEEE Trans. Instrum. Meas. **67**, 1994-2000 (2018) [25]	Optical	Corrugated silver membrane	1414*	86.97
Opt. Express **29**, 16447-16454 (2021) [17]	Optical	Glass/No membrane	282.3*	530
Opt. Lett. **45**, 3516-3519 (2020) [18]	Optical	Polymer/Spiral	167.3	0.33
Nano Lett. **19**, 2627-2633 (2019) [16]	Optical	Biomimetic	15*	1665
Microsyst. Nanoeng. **9**, 65 (2023) [19]	Optical	CaF_2 microring	11540*	9.4
Opt. Express **31**, 21796-21805 (2023) [15]	Optical	Steel cantilever	1058*	6.2
Sens. Actuators, A **279**, 107-112 (2018) [29]	Optical	Steel cantilever	174.6	8.5
**This work**	Optical	Diamond cantilever	51500*	0.018

* Sensitivity values were reported at resonance frequencies

References

[1] K. Ma, *et al.*, A wave-confining metasphere beamforming acoustic sensor for superior human-machine voice interaction. *Science Advances* **8** (39), eadc9230 (2022).

[2] A. Boisen, S. Dohn, S. S. Keller, S. Schmid, M. Tenje, Cantilever-like micromechanical sensors. *Reports on Progress in Physics* **74** (3), 036101 (2011).

[3] M. Héritier, *et al.*, Nanoladder cantilevers made from diamond and silicon. *Nano letters* **18** (3), 1814–1818 (2018).

[4] V. Koskinen, J. Fonsen, K. Roth, J. Kauppinen, Progress in cantilever enhanced photoacoustic spectroscopy. *Vibrational spectroscopy* **48** (1), 16–21 (2008).

[5] J. A. Bucaro, N. Lagakos, B. H. Houston, J. Jarzynski, M. Zalalutdinov, Miniature, high performance, low-cost fiber optic microphone. *The Journal of the Acoustical Society of America* **118** (3), 1406–1413 (2005).

[6] Y. Wang, *et al.*, A lever-type PDMS flexible cavity for acoustic vector sensor with high sensitivity. *IEEE Sensors Journal* **23** (6), 5637–5642 (2023).
